# Peripheral Blood-Based Biopsy for Breast Cancer Risk Prediction and Early Detection

**DOI:** 10.3389/fmed.2020.00028

**Published:** 2020-02-11

**Authors:** Farah J. Nassar, Ghada Chamandi, Mohamad Ali Tfaily, Nathalie Khoueiry Zgheib, Rihab Nasr

**Affiliations:** ^1^Department of Internal Medicine, Faculty of Medicine, American University of Beirut, Beirut, Lebanon; ^2^Department of Anatomy, Cell Biology and Physiological Sciences, Faculty of Medicine, American University of Beirut, Beirut, Lebanon; ^3^Department of Pharmacology and Toxicology, Faculty of Medicine, American University of Beirut, Beirut, Lebanon

**Keywords:** breast cancer, liquid biopsy, early detection, risk prediction, microRNA, cfDNA, exosome, methylation patterns

## Abstract

Among women, breast cancer (BC) is not only the most common cancer worldwide but also the leading cause of cancer death. Only 5–10% of breast cancer cases are attributed to inherited mutations (BRCA1, BRCA2, and other breast cancer susceptibility genes). Breast cancer incidence has been rising particularly in young women who are not eligible for mammography, and it has been acting as a burden especially in developing countries that lack screening and awareness programs. For this reason, research has shifted to use minimally invasive liquid biopsies especially blood-based biomarkers with potential value for breast cancer risk prediction and early detection. This mini-review will tackle the different blood-based biomarkers focusing mainly on circulating miRNA, circulating proteins, cell-free nucleic acids, methylation patterns, and exosomes. It also introduces the potential opportunities for the clinical use of these blood-based biomarkers for breast cancer risk prediction.

## Introduction

Breast cancer (BC) is the second most common cancer worldwide, with an incidence and mortality of 2,088,849 and 626,679, respectively in 2018. These alarming numbers are expected to continue rising by the year 2040 (1), hence the need to develop newer strategies for early detection and predisposition to BC. Predisposition to BC is not solely dependent on one risk factor; thus several BC risk assessment models were developed for that purpose. Regarding early detection, several randomized trials showed that screening can decrease BC burden and mortality, with a 0.74 relative risk of mortality among women who underwent mammography compared to those who did not, particularly for the age groups between 50 and 74 years (2–4). The selection of screening age depends on the age of BC onset in each population as well as the poor sensitivity of this screening method before the age of 40 (5, 6). Notably, the median age of BC diagnosis in developing countries remains a decade lower than that of Western Europe and the United States, which is 62 years (7, 8). For example, 70% of BC patients in Sub-Saharan Africa present with BC before the age of 50 years, making mammography a poor screening tool for the majority of this population (8–10). In addition to that, mammography can cause discomfort, overdiagnosis, and false-positive results accompanied by patient distress and anxiety (6). Imaging based diagnostic tools are also expensive and may not have the same performance and quality everywhere as well as may not be available equally for all populations especially people residing in rural areas.

Therefore, investigators shifted their scientific focus toward developing novel minimally invasive methods for early BC detection and risk prediction. Recently, liquid biopsy is the measurement of markers from easily accessible biologic fluids such as saliva, urine, and peripheral blood has become an attractive and increasingly investigated field of research. It was first introduced by Diaz et al. (11) in 2014 for the detection and examination of circulating tumor DNA in the blood. Then, its use was extended for the analysis of other circulating biomarkers such as microRNAs, exosomes, cell-free DNA, proteins, and methylated genes. There has been accumulating evidence for the potential clinical value of peripheral blood-based biopsy for cancer risk prediction and diagnosis, tracking of disease relapse and resistance, and stratification of patients for targeted therapy.

**Figure 1:**
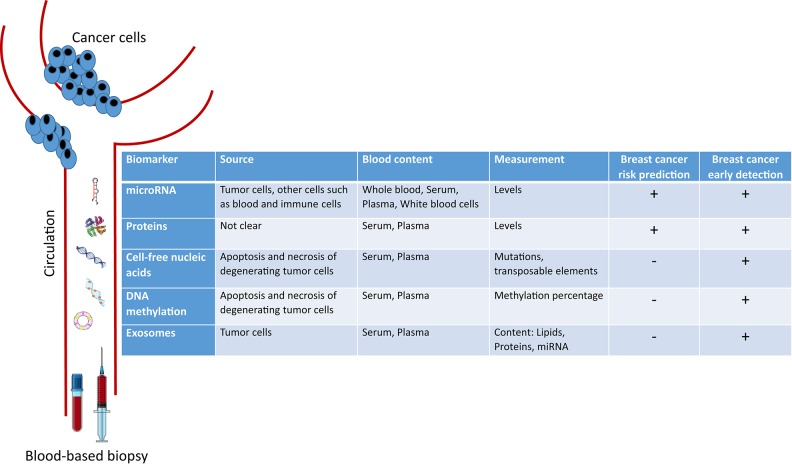
Peripheral blood-based biopsy for breast cancer risk prediction and early detection.

In this mini-review, we introduce the novel circulating blood-based biomarkers that are being investigated for either early BC detection or risk prediction. We focus on circulating microRNAs, proteins, cell-free nucleic acids, DNA methylation patterns, and exosomes (Figure 1).

### Methodology

The research strategy for this review was guided by the main objective of reviewing the role of peripheral blood-based biopsy for BC risk prediction and early detection. The guiding specific question was: what empirical research is available on specific blood-based biomarkers in BC? This comprehensive research strategy targeted mainly journal articles published in English with no year specification. Only PubMed database was used with the following MeSH (Medical Subjects Headings) key terms.

(1) ***breast neoplasms*** *AND* ***microRNA***
(2) ***breast neoplasms*** *AND* ***circulating*** *AND* ***protein***
(3) ***breast neoplasms*** *AND* ***circulating*** *AND* ***DNA/RNA/lnRNA***
(4) ***breast neoplasms*** *AND* ***circulating*** *AND* ***DNA*** *AND* ***methylation***
(5) ***breast neoplasms*** *AND* ***exosomes***.

Following this, the compiled abstracts were discussed among the research team. Only articles that were on human samples and concerned with BC risk prediction and early detection were exported to EndNote software.

## Circulating microRNA

microRNAs (miRNA) are small non-coding RNA that regulate gene expression at the post-transcriptional level (12). miRNAs can act as oncogenes or tumor suppressors; thus playing an important role in tumor pathogenesis (13). As such, different miRNAs were shown to be dysregulated in cancer tissues, especially in BC as compared to normal tissues (14, 15). Moreover, miRNA dysregulation may be reflected in the biological fluids of BC patients including serum, plasma, and whole blood. miRNA are easily quantifiable, stable and resistant to degradation in the extracellular environment, hence supporting their potential role as biomarkers for BC screening and diagnosis (16, 17).

Dysregulation of circulating miRNA was noted in women who were at risk of developing BC. miR-144-3p, miR-451a, and miR-144-5p were found to be upregulated, while miR-708-5p was found to be downregulated in prospectively collected PBMC of 20 women who were unaffected at the time of recruitment and later diagnosed with breast cancer, as compared to 20 unaffected control women. However, these results failed to be confirmed using quantitative reverse transcription polymerase chain reaction (RT-PCR) in a validation set (18). Another study worth noting found that miR-195-5p and miR-495 are downregulated in PBMC of BC patients compared to healthy subjects, with a 77.8 and 100% sensitivity and 100 and 66.7% specificity, respectively, enabling them to be valuable diagnostic tools (19). It was not until 2009 when Zhu et al. (20) demonstrated that miRNA deregulation can also be detected in the serum of BC patients. In a following prospective cohort study on 205 cases of BC matched with 205 controls from the Sister Study Cohort with all recruited women BC free at the time of enrollment, global miRNA expression patterns revealed 21 differentially expressed miRNAs in the serum of BC patients when compared to healthy subjects (21). Several of these dysregulated miRNA such as the downregulated miR-99a-5p (22), miR-4634, miR-6875-5p (23), miR-18a, and miR-139-5p (24) or the upregulated miR-1246, miR-1307-3p, miR-6861-5p (23), and miR-21 (22) were later validated to be promising serum biomarkers for BC detection. In a meta-analysis by Li et al. (25), diagnosing BC by measuring serum miR-21 levels were found to be associated with high sensitivity and specificity of 0.79 and 0.85, respectively.

Even though several candidate miRNAs were individually studied as potential biomarkers for BC detection, they all failed to replace currently available detection models. This is due to the absence of standardization in the pre-analytical variables such as sample processing, storage, and handling, as well as data normalization strategy for miRNA quantification. This led several investigators to assess early detection with combinations of different miRNAs in the body fluids, an endeavor that translated into promising results in terms of sensitivity and specificity. For example, a study showed that selected miRNA signatures (such as in miR-21-3p, miR-21-5p, and miR-99a-5p) from miRNA profiles of 409 early breast cancer patients and 87 healthy controls from The Cancer Genome Atlas database were successfully validated as serum miRNA signatures with a diagnostic sensitivity and specificity of 97.9 and 73.5%, respectively (22). Also, a large cohort study investigated a combination of five miRNAs (miR-1246, miR-1307-3p, miR-4634, miR-6861-5p, and miR-6875-5p) in sera of 1,206 BC patients using microarray for expression analysis and quantitative RT-PCR for validation. This combination was shown to have a sensitivity of 97.3%, a specificity of 82.9%, and an accuracy of 89.7%, with the potential to detect early BC and to differentiate it from other possible tumors (23). As for plasma, other combinations of miRNAs were also able to detect BC with high sensitivity (26). These combinations included miR-192-5p/miR-382-5p and miR-192-5p/miR-574-5p (26).

Besides their growing role in early detection of BC, miRNAs have been evaluated as potential circulating biomarkers to predict BC risk. As such, a study measured serum miRNA deregulation in 48 patients at high risk of developing BC, 24 of whom eventually developed the disease. A panel of 6 miRNA showed an ability to predict the risk of BC with high accuracy and precision (27). Nevertheless, and despite these encouraging results, more studies are needed to investigate circulating miRNA's role in BC risk prediction.

## Circulating Proteins

Several tumor proteins are detected in circulation though their origin is not known; however, only a few of them were shown to be clinically useful biomarkers in BC. The most currently measured circulating tumor protein markers are Carcinoembryonic antigen (CEA) and Cancer antigen (CA) 15-3 (also known as MUC1). These are however more useful for assessment of BC prognosis and recurrence rather than diagnosis since they lack specificity and sensitivity for low-volume disease (28, 29).

Recently, 8-hydroxy-2'-deoxyguanosine (8-OHdG), a nucleic acid damage marker due to oxidative stress, was reported to be a potential circulating biomarker for early detection of BC by two studies conducted on two different ethnic groups (Spain and Saudi Arabia). For instance, blood levels of 8-OHdG were significantly higher in women with BC group as compared to healthy women. The same pattern of 8-OHdG was observed with another diagnostic marker, which is cancer antigen CA 15-3 (30, 31). Moreover, in a prospective study including 2,835 cases and 3,122 matched controls from 10 cohorts, circulating anti-Müllerian hormone that is usually produced by ovaries also correlated with BC risk, particularly with ER+/PR+ tumors, with a 60% higher risk for women in the top quartile as compared to the bottom quartile of anti-Müllerian blood concentration (32).

Other circulating proteins under active investigation include the circulating adipose fatty acid-binding protein (A-FABP) that was recently shown to promote the development of BC in obese patients (33). Also, adipose metabolism has been linked to BC risk as plasma concentrations of adipose-derived fatty acid-binding protein 4 (FABP4) were found to be higher in 98 BC patients when compared to 96 healthy controls (34). Other protein regulators involved in bone resorption such as the Receptor Activator of NF-kB Ligand (RANKL), its receptor RANK, and the natural antagonist osteoprotegerin (OPG) were also found to be involved in BC (35–37). For instance, high serum levels of RANKL and RANKL/OPG ratios were reported in postmenopausal women at high risk for BC (38). Another study identified high serum OPG levels to be mainly associated with increased risk for ER- BC (39). On the other hand, a large scale investigation with a cohort of 1,976 incident invasive BC cases, of which 1,598 were ER+, showed limited evidence for correlating circulating RANKL levels with BC risk (40). Notably, and despite the availability of a myriad of BC studies in the proteomics literature (41, 42), the field is still lacking invalidated protein markers for both BC risk prediction and early detection.

## Cell-Free Nucleic Acids

In 1977, cell-free DNA (cfDNA) was first reported in the serum of cancer patients after surgery and/or chemotherapy, and its concentration varied depending on the response to therapy (43). Later in 1989, a detectable amount of cfDNA was found in the plasma of cancer patients as compared to that of normal subjects (44). The origin of this extracellular DNA was shown to be mainly from the apoptosis and necrosis of degenerating cells in tumor tissue (45). cfDNA could be analyzed for specific genetic alterations including microsatellite alterations, allelic imbalance, translocations, mutations, and presence of viral genes (46, 47).

*PIK3CA* is the most commonly evaluated mutation detected in BC and occurring at a frequency of 20–45%. For instance, a prospective study assessed cfDNA *PIK3CA* mutations in the plasma of early BC patients before and after breast surgery and detected *PIK3CA* mutations preoperatively with 93.3% sensitivity and 100% specificity (48). Also, a meta-analysis that evaluated the overall diagnostic performance of cfDNA for *PIK3CA* mutation detection in BC from five different studies concluded that cfDNA *PIK3CA* mutation has a pooled sensitivity and specificity of 86 and 98%, respectively, with highest diagnostic accuracy in metastatic BC (49). More recently, next-generation sequencing of cfDNA in plasma of 100 women pretreated for advanced BC revealed the presence of a landscape of somatic mutations in different genes, such as *TP53, PIK3CA, ESR1*, and *NOTCH1*, in different subtypes of advanced BC (50). These results underscore the fact that BC is a heterogeneous disease, hence several mutations could be present, and researchers ought to analyze combinations of multiple cDNA targets.

Other recently studied cfDNA biomarkers for early BC detection are *LINE1* and *ALU*. These are transposable elements that were referred to as “junk DNA” in the past. A pilot study showed that *LINE1* copy number is significantly higher in the serum of 36 BC patients as compared to 29 healthy subjects (51). Similarly, serum *ALU115* levels and *ALU247/115* index or ratio were significantly higher in 40 patients newly diagnosed with BC patients as compared to 40 healthy controls. Serum *ALU247/115* index or ratio was the best in terms of sensitivity, specificity, positive and negative prediction values, and total efficiency of BC diagnosis when compared to *ALU115* levels and serum concentration of CEA and CA15 proteins. Notably, that improved sensitivity (97.5%) and negative prediction values (96.4%) were attained when all of the latter biomarkers were combined (52). Another study identified plasma cfDNA *ALU-247, ALU-115*, and DNA integrity (ratio between *ALU 247* and *115*) as potential biomarkers for BC diagnosis upon evaluating them in 40 BC patients and 10 healthy volunteers (53).

In addition to DNA, cfRNA can be found in the circulation. For example, long non-coding RNA (lncRNA), has also been examined as a potential biomarker for BC early detection. As such, large intergenic non-coding RNA-ROR (lncROR) measured in 96 plasma samples from BC patients had a high sensitivity (80.0%) and specificity (73.3%) for BC detection, and these values were greater than those of CEA and CA15-3 measured from the same patients (54). Similarly, two other lncRNA, H19, and HOX transcript antisense intergenic ribonucleic acid (HOTAIR), were identified as promising markers for BC detection in plasma (55, 56).

## Circulating DNA Methylation Patterns

DNA methylation is one of the hallmarks of epigenetic modifications associated with cancer. Several studies on DNA methylation in cancer have utilized cell-free DNA from plasma and serum to assess differences in methylation levels between BC patients and healthy controls (57). For example, significant DNA hypermethylation of *APC* and *RAR*β_2_ were detected in the serum of patients with malignant BC as compared to serum from subjects with benign lesions and healthy controls, with both sensitivities and specificities of these two methylated genes being superior to traditional tumor markers (CEA and CA 15-3) for BC detection (58). Another study revealed that the hypermethylation of at least one of these genes (*APC, GSTP1, RASSF1A*, and *RAR*β*2*) can be detected with a sensitivity of 62% and a specificity of 87% in BC (59). Another study examined the promoter methylation of six genes, *SFN, P16, hMLH1, HOXD13, PCDHGB7*, and *RASSF1a* in the serum of 749 subjects including patients with BC, patients with benign breast diseases, and healthy women. Results indicated that methylation analysis of the six-gene panel had significantly high sensitivities of 82.4 and 79.6% and specificities of 78.1 and 72.4% in the diagnosis of BC when compared to subjects with benign disease and healthy controls, respectively (60). In contrast, a recent paper showed that there were no significant differences in the levels of methylation of *RASSF1a* and *ATM* in peripheral blood DNA of 229 sporadic BC patients compared to that of 151 healthy controls (61). Other investigators evaluated DNA methylation of *14-3-3* σ promoter in circulation and produced controversial results (62, 63). Results from all of the above-described studies highlight the fact that the measurement of circulating DNA methylation patterns requires further investigation before being translated to clinical practice in BC (57).

## Circulating Exosomes

Exosomes are membrane-derived nanoscale vesicles that are actively released by most cells into the circulation (64). The content of these tiny particles, which are also shed by cancer cells, includes DNA, lipids, messenger RNA, microRNA, and other small regulatory RNA. Relevant molecular information can be obtained by analyzing exosomes' content. Exosomes and their cargo have been shown to play an important role in cell-cell communication between the tumor and the stroma, and in establishing the pre-metastatic niche. They demonstrate a promising blood-based biomarker for early cancer detection (65–67), as well as for BC since much higher levels of exosomes with altered cargo were found in sera of BC patients relative to healthy subjects (68).

It was also reported that exosomes released by BC cells into biological fluids contain important information about the primary tumor (69). For example, miRNA-containing exosomes (Exo-miR), an important and abundant exosomal cargo, were shown to potentially represent an ideal biomarker of disease onset (70, 71). As such, the diagnostic value of serum exosomal miRNA in BC was studied (72), nevertheless, no exosomal analysis was reported in subjects with a high risk of developing cancers. However, Exo-miR-233-3p was able to discriminate between ductal carcinoma *in situ* and infiltrating ductal cancer, suggesting its potential role for the early detection of invasive BC (73). Moreover, exosomal miR-21 and miR-1246 were found to be higher in plasma of BC patients or mice transplanted with patients derived breast tumors as compared to healthy controls (74). Furthermore, there exists a differential expression of exosomal miR based on the tumor molecular subtypes. For instance, higher levels of exosomal miR-373 were indicative of triple-negative BC (75). In addition to miRNAs, the exosomal proteins fibronectin and developmental endothelial Locus-1 (Del-1) are promising biomarkers for early-stage BC (76, 77). Although circulating exosomes have emerged as potential candidates for a non-invasive biomarker for BC, recent efforts have focused on the detection of metastasis and assessment of disease prognosis as well as on optimizing their isolation. Few promising exo-miR candidates for early detection were reported (71). However, until now, there is no compelling evidence for the potential clinical utility of exosomes for BC risk assessment.

## Conclusions

In order to identify BC predisposition of healthy subjects, numerous BC risk prediction tools that take into consideration multiple risk factors are available (78, 79). Yet, only a few examples of peripheral blood-based biopsy have been evaluated for BC risk assessment. As for BC screening and early detection, several blood-based biomarkers are likely to be clinically used as easily accessible and minimally invasive substitutes or supplements to routine screening tests such as mammography. A comparison in the sensitivity and specificity of various blood- and serum-based biomarkers to imaging methods in the diagnosing and screening for breast cancer is required. Based on the literature (Table 1), several biomarkers have better sensitivity and specificity than imaging-based methods. For instance, miR-495 alone, a miRNA panel of miR-21-3p, miR-21-5p, and miR-99a-5p, a miRNA panel of miR-1246, miR-1307-3p, miR-4634, miR-6861-5p, and miR-6875-5p, PIK3CA proteins, ALU115 combined with ALU247/115 cfDNA, CEA, and CA15-3, methylated APC and RARβ2, as well as Del-1 exosomes, appear to have the highest sensitivities, even as compared to the current imaging screening standards, making them potential screening tool for early breast cancer. On the other hand, miR-195-5p, mutated PIK3CA, and methylated APC and RARβ2 and 14-3-3 σ promoter have the highest specificities. This makes them powerful diagnostic tools for breast cancer, especially for PIK3CA protein, and methylated 14-3-3 σ promoter as the evidence is based on meta-analyses. Further studies and meta-analyses are needed to provide stronger evidence for these data before adopting these biomarkers for screening and early detection of breast cancer.

**Table 1:** Sensitivities and specificities of different breast cancer detection methods (Imaging and blood-based biomarkers).

**Detection method**	**Biomarker**	**Sensitivity %**	**Specificity %**	**Meta-analysis Y/N**	**References**
Imaging	Mammography	89	84	Y	(80)
	MRI	90	72	Y	(81)
	Ultrasound	80.1	88.4	Y	(82)
microRNA	miR-21	79	85	Y	(25)
	miR-195-5p	77.8	100	N	(19)
	miR-495	100	66.7	N	(19)
	miR-1246, miR-1307-3p, miR-4634, miR-6861-5p, and miR-6875-5p	97.3	82.9	N	(23)
	miR-21-3p, miR-21-5p, and miR-99a-5p	97.9	73.5	N	(22)
	miR-21-3, miR-192-5p, miR-221-3p, miR-451a, miR-574-5p, miR-1273g-3p, hsa-miR-152, miR-22-3p, miR-222-3p, miR-30a-5p, miR-30e-5p, miR-324-3p, and miR-382-5p	88.1	77.5	N	(26)
Proteins	8-OHdG	82	80	N	(31)
Cell free nucleic acids	cfDNA concentration	87	87	Y	(83)
	*PIK3CA*	86	98	Y	(49)
	ALU115, ALU247/115, CEA, and CA15-3	97.5	67.5	N	(52)
	lncROR	80	73.3	N	(54)
	H19	56.7	86.7	N	(56)
	HOTAIR	80	68.3	N	(55)
DNA methylation	*APC*	93.4	95.4	N	(58)
	*RARβ_2_*	95.5	92.4	N	(58)
	*APC, GSTP1, RASSF1A*, and *RARβ2*	62	87	N	(59)
	*SFN, P16, hMLH1, HOXD13, PCDHGB7, and RASSF1a*	79.6	72.4	N	(60)
	14-3-3 σ promoter	69	99	Y	(63)
Exosomes	Del-1	94.7	86.36	N	(77)
	FN	69.2	73.3	N	(76)

The field of liquid biopsy research is still in its infancy but it is evolving rapidly and providing a rich space for discovery. To speed up the process of discovery and clinical translation, research should resolve some of the overarching challenges. Most of the studies on blood based biomarkers are retrospective case-control with a small sample size and with variable methodologies of sample handling and storage. Hence, studies should examine biomarkers in large ethnically diverse populations as well as prospectively measuring levels in healthy subjects especially those with a high risk of developing cancer well before the appearance of symptoms. Furthermore, the deficiency of standardized and robust methods for sample isolation, quantification and analysis, and the lack of benchmarking the sensitivity and specificity of biomarkers in large and ethnically diverse BC cohorts in comparison not only to healthy subjects but also to other cancer patients (84). By overcoming these drawbacks, the clinical application of these small molecules will surely amaze the world and save lives due to more accurate risk prediction and earlier detection of BC.

## Author Contributions

All authors contributed to the writing and critical reviewing of the article.

### Conflict of Interest

The authors declare that the research was conducted in the absence of any commercial or financial relationships that could be construed as a potential conflict of interest.

## References

[1] Bray F Ferlay J Soerjomataram I Siegel RL Torre LA Jemal A. Global cancer statistics 2018: GLOBOCAN estimates of incidence and mortality worldwide for 36 cancers in 185 countries. CA Cancer J Clin. (2018) 68:394–424. 10.3322/caac.21492 30207593

[2] Tabár L Gad A Holmberg LH Ljungquist U Fagerberg CJG Baldetorp L Reduction in mortality from breast cancer after mass screening with mammography: randomised trial from the Breast Cancer Screening Working Group of the Swedish National Board of Health and Welfare. Lancet. (1985) 325:829–32. 10.1016/S0140-6736(85)92204-4 2858707

[3] Kerlikowske K Grady D Rubin SM Sandrock C Ernster VL. Efficacy of screening mammography: a meta-analysis. JAMA. (1995) 273:149–54. 10.1001/jama.1995.03520260071035 7799496

[4] Ajai A 16-year mortality from breast cancer in the UK trial of early detection of breast cancer. Lancet. (1999) 353:1909–14. 10.1016/S0140-6736(98)07412-1 10371568

[5] Saslow D Boetes C Burke W Harms S Leach MO Lehman CD. American Cancer Society Guidelines for breast screening with MRI as an adjunct to mammography. CA Cancer J Clin. (2007) 57:75–89. 10.3322/canjclin.57.2.75 17392385

[6] Lauby-Secretan B Scoccianti C Loomis D Benbrahim-Tallaa L Bouvard V Bianchini F. Breast-cancer screening — viewpoint of the IARC Working Group. N Engl J Med. (2015) 372:2353–8. 10.1056/NEJMsr1504363 26039523

[7] Najjar H Easson A. Age at diagnosis of breast cancer in Arab nations. Int J Surg. (2010) 8:448–52. 10.1016/j.ijsu.2010.05.012 20601253

[8] Sighoko D Kamate B Traore C Malle B Coulibaly B Karidiatou A. Breast cancer in pre-menopausal women in West Africa: analysis of temporal trends and evaluation of risk factors associated with reproductive life. Breast. (2013) 22:828–35. 10.1016/j.breast.2013.02.011 23489760

[9] Sighoko D Bah E Haukka J McCormack VA Aka EP Bourgeois D. Population-based breast (female) and cervix cancer rates in the Gambia: evidence of ethnicity-related variations. Int J Cancer. (2010) 127:2248–56. 10.1002/ijc.25244 20162609

[10] Jedy-Agba E Curado MP Ogunbiyi O Oga E Fabowale T Igbinoba F. Cancer incidence in Nigeria: a report from population-based cancer registries. Cancer Epidemiol. (2012) 36:e271–8. 10.1016/j.canep.2012.04.007 22621842 PMC3438369

[11] Diaz LA Jr Bardelli A. Liquid biopsies: genotyping circulating tumor DNA. J Clin Oncol. (2014) 32:579–86. 10.1200/JCO.2012.45.2011 24449238 PMC4820760

[12] Lee RC Feinbaum RL Ambros V. The *C. elegans* heterochronic gene lin-4 encodes small RNAs with antisense complementarity to lin-14. Cell. (1993) 75:843–54. 10.1016/0092-8674(93)90529-Y 8252621

[13] Peng Y Croce CM. The role of microRNAs in human cancer. Signal Transduct Target Ther. (2016) 1:15004. 10.1038/sigtrans.2015.4 29263891 PMC5661652

[14] Iorio MV Ferracin M Liu C-G Veronese A Spizzo R Sabbioni S. microRNA gene expression deregulation in human breast cancer. Cancer Res. (2005) 65:7065–70. 10.1158/0008-5472.CAN-05-1783 16103053

[15] Pigati L Yaddanapudi SC Iyengar R Kim DJ Hearn SA Danforth D. Selective release of microRNA species from normal and malignant mammary epithelial cells. PLoS ONE. (2010) 5:e13515. 10.1371/journal.pone.0013515 20976003 PMC2958125

[16] Weber JA Baxter DH Zhang S Huang DY How Huang K Jen Lee M. The microRNA spectrum in 12 body fluids. Clin Chem. (2010) 56:1733–41. 10.1373/clinchem.2010.147405 20847327 PMC4846276

[17] Sohel MH Extracellular/circulating microRNAs: release mechanisms, functions, and challenges. Achiev Life Sci. (2016) 10:175–86. 10.1016/j.als.2016.11.007

[18] Chang CW Wu HC Terry MB Santella RM. microRNA expression in prospectively collected blood as a potential biomarker of breast cancer risk in the BCFR. Anticancer Res. (2015) 35:3969–77. 26124344 PMC4776637

[19] Mishra S Srivastava AK Suman S Kumar V Shukla Y. Circulating miRNAs revealed as surrogate molecular signatures for the early detection of breast cancer. Cancer Lett. (2015) 369:67–75. 10.1016/j.canlet.2015.07.045 26276721

[20] Zhu W Qin W Atasoy U Sauter ER. Circulating microRNAs in breast cancer and healthy subjects. BMC Res Notes. (2009) 2:89. 10.1186/1756-0500-2-89 19454029 PMC2694820

[21] Godfrey AC Xu Z Weinberg CR Getts RC Wade PA DeRoo LA. Serum microRNA expression as an early marker for breast cancer risk in prospectively collected samples from the Sister Study cohort. Breast Cancer Res. (2013) 15:R42. 10.1186/bcr3428 23705859 PMC3706791

[22] Yu X Liang J Xu J Li X Xing S Li H. Identification and validation of circulating microRNA signatures for breast cancer early detection based on large scale tissue-derived data. J Breast Cancer. (2018) 21:363–70. 10.4048/jbc.2018.21.e56 30607157 PMC6310725

[23] Shimomura A Shiino S Kawauchi J Takizawa S Sakamoto H Matsuzaki J. Novel combination of serum microRNA for detecting breast cancer in the early stage. Cancer Sci. (2016) 107:326–34. 10.1111/cas.12880 26749252 PMC4814263

[24] Kodahl AR Lyng MB Binder H Cold S Gravgaard K Knoop AS. Novel circulating microRNA signature as a potential non-invasive multi-marker test in ER-positive early-stage breast cancer: a case control study. Mol Oncol. (2014) 8:874–83. 10.1016/j.molonc.2014.03.002 24694649 PMC5528529

[25] Li S Yang X Yang J Zhen J Zhang D. Serum microRNA-21 as a potential diagnostic biomarker for breast cancer: a systematic review and meta-analysis. Clin Exp Med. (2016) 16:29–35. 10.1007/s10238-014-0332-3 25516467

[26] Fang R Zhu Y Hu L Khadka VS Ai J Zou H. Plasma microRNA pair panels as novel biomarkers for detection of early stage breast cancer. Front Physiol. (2019) 9:1879. 10.3389/fphys.2018.01879 30670982 PMC6331533

[27] Farina NH Ramsey JE Cuke ME Ahern TP Shirley DJ Stein JL. Development of a predictive miRNA signature for breast cancer risk among high-risk women. Oncotarget. (2017) 8:112170–83. 10.18632/oncotarget.22750 29348816 PMC5762501

[28] Sławicki S Mroczko B Szmitkowski M. Tumor markers of breast cancer. Postepy Higieny I Medycyny Doswiadczalnej. (2004) 58:292–300. 15280799

[29] Kabel AM Tumor markers of breast cancer: new prospectives. J Oncol Sci. (2017) 3:5–11. 10.1016/j.jons.2017.01.001

[30] Bayo J Castano MA Rivera F Navarro F. Analysis of blood markers for early breast cancer diagnosis. Clin Transl Oncol. (2018) 20:467–75. 10.1007/s12094-017-1731-1 28808872

[31] Nour Eldin EEM El-Readi MZ Nour Eldein MM Alfalki AA Althubiti MA Mohamed Kamel HF. 8-hydroxy-2'-deoxyguanosine as a discriminatory biomarker for early detection of breast cancer. Clin Breast Cancer. (2019) 19:e385–93. 10.1016/j.clbc.2018.12.013 30683611

[32] Ge W Clendenen TV Afanasyeva Y Koenig KL Agnoli C Brinton LA. Circulating anti-Mullerian hormone and breast cancer risk: a study in ten prospective cohorts. Int J Cancer. (2018) 142:2215–26. 10.1002/ijc.31249 29315564 PMC5922424

[33] Hao J Zhang Y Yan X Yan F Sun Y Zeng J. Circulating adipose fatty acid binding protein is a new link underlying obesity-associated breast/mammary tumor development. Cell Metab. (2018) 28:689–705.e685. 10.1016/j.cmet.2018.07.006 30100196 PMC6221972

[34] Guaita-Esteruelas S Saavedra-Garcia P Bosquet A Borras J Girona J Amiliano K. Adipose-derived fatty acid-binding proteins plasma concentrations are increased in breast cancer patients. Oncologist. (2017) 22:1309–15. 10.1634/theoncologist.2016-0483 28701570 PMC5679823

[35] Schramek D Leibbrandt A Sigl V Kenner L Pospisilik JA Lee HJ. Osteoclast differentiation factor RANKL controls development of progestin-driven mammary cancer. Nature. (2010) 468:98–102. 10.1038/nature09387 20881962 PMC3084017

[36] Nolan E Vaillant F Branstetter D Pal B Giner G Whitehead L. RANK ligand as a potential target for breast cancer prevention in BRCA1-mutation carriers. Nat Med. (2016) 22:933–9. 10.1038/nm.4118 27322743

[37] Sigl V Owusu-Boaitey K Joshi PA Kavirayani A Wirnsberger G Novatchkova M. RANKL/RANK control Brca1 mutation. Cell Res. (2016) 26:761–74. 10.1038/cr.2016.69 27241552 PMC5129883

[38] Kiechl S Schramek D Widschwendter M Fourkala EO Zaikin A Jones A. Aberrant regulation of RANKL/OPG in women at high risk of developing breast cancer. Oncotarget. (2017) 8:3811–25. 10.18632/oncotarget.14013 28002811 PMC5354797

[39] Fortner RT Sarink D Schock H Johnson T Tjonneland A Olsen A. Osteoprotegerin and breast cancer risk by hormone receptor subtype: a nested case-control study in the EPIC cohort. BMC Med. (2017) 15:26. 10.1186/s12916-017-0786-8 28173834 PMC5297136

[40] Sarink D Schock H Johnson T Chang-Claude J Overvad K Olsen A. Receptor activator of nuclear factor kB ligand, osteoprotegerin, and risk of death following a breast cancer diagnosis: results from the EPIC cohort. BMC Cancer. (2018) 18:1010. 10.1186/s12885-018-4887-3 30348163 PMC6196438

[41] Hathout Y. Proteomic methods for biomarker discovery and validation. Are we there yet? Exp Rev Proteom. (2015) 12:329–31. 10.1586/14789450.2015.1064771 26186709

[42] Boschetti E D'Amato A Candiano G Righetti PG. Protein biomarkers for early detection of diseases: the decisive contribution of combinatorial peptide ligand libraries. J Proteom. (2018) 188:1–14. 10.1016/j.jprot.2017.08.009 28882677

[43] Leon SA Shapiro B Sklaroff DM Yaros MJ. Free DNA in the serum of cancer patients and the effect of therapy. Cancer Res. (1977) 37:646–50. 837366

[44] Stroun M Anker P Maurice P Lyautey J Lederrey C Beljanski M. Neoplastic characteristics of the DNA found in the plasma of cancer patients. Oncology. (1989) 46:318–22. 10.1159/000226740 2779946

[45] Jahr S Hentze H Englisch S Hardt D Fackelmayer FO Hesch R-D. DNA Fragments in the blood plasma of cancer patients: quantitations and evidence for their origin from apoptotic and necrotic cells. Cancer Res. (2001) 61:1659–65. 11245480

[46] Chen XQ Stroun M Magnenat JL Nicod LP Kurt AM Lyautey J. Microsatellite alterations in plasma DNA of small cell lung cancer patients. Nat Med. (1996) 2:1033–5. 10.1038/nm0996-1033 8782463

[47] Chang HW Lee SM Goodman SN Singer G Cho SK Sokoll LJ. Assessment of plasma DNA levels, allelic imbalance, and CA 125 as diagnostic tests for cancer. J Natl Cancer Inst. (2002) 94:1697–703. 10.1093/jnci/94.22.1697 12441325

[48] Beaver JA Jelovac D Balukrishna S Cochran R Croessmann S Zabransky DJ. Detection of cancer DNA in plasma of patients with early-stage breast cancer. Clin Cancer Res. (2014) 20:2643–50. 10.1158/1078-0432.CCR-13-2933 24504125 PMC4024333

[49] Zhou Y Wang C Zhu H Lin Y Pan B Zhang X. Diagnostic Accuracy of PIK3CA mutation detection by circulating free DNA in breast cancer: a meta-analysis of diagnostic test accuracy. PLoS ONE. (2016) 11:e0158143. 10.1371/journal.pone.0158143 27336598 PMC4918940

[50] Yi Z Ma F Li C Chen R Yuan L Sun X. Landscape of somatic mutations in different subtypes of advanced breast cancer with circulating tumor DNA analysis. Sci Rep. (2017) 7:5995. 10.1038/s41598-017-06327-4 28729728 PMC5519668

[51] Sunami E Vu AT Nguyen SL Giuliano AE Hoon DS. Quantification of LINE1 in circulating DNA as a molecular biomarker of breast cancer. Ann N Y Acad Sci. (2008) 1137:171–4. 10.1196/annals.1448.011 18837943

[52] Tang Z Li L Shen L Shen X Ju S Cong H. Diagnostic value of serum concentration and integrity of circulating cell-free DNA in breast cancer: a comparative study with CEA and CA15–3. Lab Med. (2018) 49:323–8. 10.1093/labmed/lmy019 29701836

[53] Hussein NA Mohamed SN Ahmed MA. Plasma ALU-247, ALU-115, and cfDNA integrity as diagnostic and prognostic biomarkers for breast cancer. Appl Biochem Biotechnol. (2018) 187:1028–45. 10.1007/s12010-018-2858-4 30151636

[54] Zhao T Wu L Li X Dai H Zhang Z. Large intergenic non-coding RNA-ROR as a potential biomarker for the diagnosis and dynamic monitoring of breast cancer. Cancer Biomark. (2017) 20:165–73. 10.3233/CBM-170064 28869448

[55] Zhang L Song X Wang X Xie Y Wang Z Xu Y. Circulating DNA of HOTAIR in serum is a novel biomarker for breast cancer. Breast Cancer Res Treat. (2015) 152:199–208. 10.1007/s10549-015-3431-2 26033707

[56] Zhang K Luo Z Zhang Y Zhang L Wu L Liu L. Circulating lncRNA H19 in plasma as a novel biomarker for breast cancer. Cancer Biomark. (2016) 17:187–94. 10.3233/CBM-160630 27540977 PMC13020493

[57] Vu TL Nguyen TT Van Thi Hong Doan LT Vo T. Methylation profiles of BRCA1, RASSF1A and GSTP1 in Vietnamese women with breast cancer. Asian Pac J Cancer Prev. (2018) 19:1887–93. 10.22034/APJCP.2018.19.7.1887 30049201 PMC6165660

[58] Swellam M Abdelmaksoud MD Mahmoud MS Ramadan A Abdel-Moneem W Hefny MM. Aberrant methylation of APC and RARβ2 genes in breast cancer patients. Iubmb Life. (2015) 67:61–8. 10.1002/iub.1346 25684670

[59] Hoque MO Feng Q Toure P Dem A Critchlow CW Hawes SE. Detection of aberrant methylation of four genes in plasma DNA for the detection of breast cancer. J Clin Oncol. (2006) 24:4262–9. 10.1200/JCO.2005.01.3516 16908936

[60] Shan M Yin H Li J Li X Wang D Su Y. Detection of aberrant methylation of a six-gene panel in serum DNA for diagnosis of breast cancer. Oncotarget. (2016) 7:18485. 10.18632/oncotarget.7608 26918343 PMC4951303

[61] Cao X Tang Q Holland-Letz T Gündert M Cuk K Schott S. Evaluation of promoter methylation of RASSF1A and ATM in peripheral blood of breast cancer patients and healthy control individuals. Int J Mol Sci. (2018) 19:900. 10.3390/ijms19030900 29562656 PMC5877761

[62] Jing F Zhang J Tao J Zhou Y Jun L Tang X. Hypermethylation of tumor suppressor genes BRCA1, p16 and 14–3-3sigma in serum of sporadic breast cancer patients. Onkologie. (2007) 30:14–9. 10.1159/000096892 17264521

[63] Ye M Huang T Ying Y Li J Yang P Ni C. Detection of 14–3-3 sigma (σ) promoter methylation as a noninvasive biomarker using blood samples for breast cancer diagnosis. Oncotarget. (2016) 8:9230–42. 10.18632/oncotarget.13992 27999208 PMC5354727

[64] Boyiadzis M Whiteside TL. Information transfer by exosomes: a new frontier in hematologic malignancies. Blood Rev. (2015) 29:281–90. 10.1016/j.blre.2015.01.004 25686749

[65] Costa-Silva B Aiello NM Ocean AJ Singh S Zhang H Thakur BK. Pancreatic cancer exosomes initiate pre-metastatic niche formation in the liver. Nat Cell Biol. (2015) 17:816–26. 10.1038/ncb3169 25985394 PMC5769922

[66] Melo SA Luecke LB Kahlert C Fernandez AF Gammon ST Kaye J. Glypican-1 identifies cancer exosomes and detects early pancreatic cancer. Nature. (2015) 523:177–82. 10.1038/nature14581 26106858 PMC4825698

[67] Wendler F Favicchio R Simon T Alifrangis C Stebbing J Giamas G. Extracellular vesicles swarm the cancer microenvironment: from tumor-stroma communication to drug intervention. Oncogene. (2017) 36:877–84. 10.1038/onc.2016.253 27546617

[68] Melo SA Sugimoto H O'Connell JT Kato N Villanueva A Vidal A. Cancer exosomes perform cell-independent microRNA biogenesis and promote tumorigenesis. Cancer Cell. (2014) 26:707–21. 10.1016/j.ccell.2014.09.005 25446899 PMC4254633

[69] Guzman N Agarwal K Asthagiri D Yu L Saji M Ringel MD. Breast cancer-specific miR signature unique to extracellular vesicles includes microRNA-like tRNA fragments. Mol Cancer Res. (2015) 13:891–901. 10.1158/1541-7786.MCR-14-0533 25722304 PMC4503213

[70] Ciesla M Skrzypek K Kozakowska M Loboda A Jozkowicz A Dulak J. microRNAs as biomarkers of disease onset. Anal Bioanal Chem. (2011) 401:2051–61. 10.1007/s00216-011-5001-8 21544542

[71] Sempere LF Keto J Fabbri M. Exosomal microRNAs in breast cancer towards diagnostic and therapeutic applications. Cancers. (2017) 9:e71. 10.3390/cancers9070071 28672799 PMC5532607

[72] Joyce DP Kerin MJ Dwyer RM. Exosome-encapsulated microRNAs as circulating biomarkers for breast cancer. Int J Cancer. (2016) 139:1443–8. 10.1002/ijc.30179 27170104

[73] Yoshikawa M Iinuma H Umemoto Y Yanagisawa T Matsumoto A Jinno H. Exosome-encapsulated microRNA-223–3p as a minimally invasive biomarker for the early detection of invasive breast cancer. Oncol Lett. (2018) 15:9584–92. 10.3892/ol.2018.8457 29805680 PMC5958689

[74] Hannafon BN Trigoso YD Calloway CL Zhao YD Lum DH Welm AL. Plasma exosome microRNAs are indicative of breast cancer. Breast Cancer Res. (2016) 18:90. 10.1186/s13058-016-0753-x 27608715 PMC5016889

[75] Eichelser C Stuckrath I Muller V Milde-Langosch K Wikman H Pantel K. Increased serum levels of circulating exosomal microRNA-373 in receptor-negative breast cancer patients. Oncotarget. (2014) 5:9650–63. 10.18632/oncotarget.2520 25333260 PMC4259427

[76] Moon PG Lee JE Cho YE Lee SJ Chae YS Jung JH. Fibronectin on circulating extracellular vesicles as a liquid biopsy to detect breast cancer. Oncotarget. (2016) 7:40189–99. 10.18632/oncotarget.9561 27250024 PMC5130002

[77] Moon PG Lee JE Cho YE Lee SJ Jung JH Chae YS. Identification of developmental endothelial locus-1 on circulating extracellular vesicles as a novel biomarker for early breast cancer detection. Clin Cancer Res. (2016) 22:1757–66. 10.1158/1078-0432.CCR-15-0654 26603257

[78] Cintolo-Gonzalez JA Braun D Blackford AL Mazzola E Acar A Plichta JK Breast cancer risk models: a comprehensive overview of existing models, validation, and clinical applications. Breast Cancer Res Treat. (2017) 164:263–84. 10.1007/s10549-017-4247-z 28444533

[79] Wood ME Farina NH Ahern TP Cuke ME Stein JL Stein GS. Towards a more precise and individualized assessment of breast cancer risk. Aging. (2019) 11:1305–16. 10.18632/aging.101803 30787204 PMC6402518

[80] Zhu X Huang JM Zhang K Xia LJ Feng L Yang P. Diagnostic value of contrast-enhanced spectral mammography for screening breast cancer: systematic review and meta-analysis. Clin Breast Cancer. (2018) 18:e985–95. 10.1016/j.clbc.2018.06.003 29983379

[81] Peters NHGM Rinkes IHMB Zuithoff NPA Mali WPTM Moons KGM Peeters PHM. Meta-analysis of MR imaging in the diagnosis of breast lesions. Radiology. (2008) 246:116–24. 10.1148/radiol.2461061298 18024435

[82] Sood R Rositch AF Shakoor D Ambinder E Pool KL Pollack E. Ultrasound for breast cancer detection globally: a systematic review and meta-analysis. J Glob Oncol. (2019) 5:1–17. 10.1200/JGO.19.00127 31454282 PMC6733207

[83] Yu D Tong Y Guo X Feng L Jiang Z Ying S. Diagnostic value of concentration of circulating cell-free DNA in breast cancer: a meta-analysis. Front Oncol. (2019) 9:95. 10.3389/fonc.2019.00095 30881916 PMC6405437

[84] Nassar FJ Nasr R Talhouk R. microRNAs as biomarkers for early breast cancer diagnosis, prognosis and therapy prediction. Pharmacol Therapeut. (2017) 172:34–49. 10.1016/j.pharmthera.2016.11.012 27916656

